# Tracing and characterization of a family outbreak of subtype B2 botulism linked to homemade pickled eggs in Jinan, China

**DOI:** 10.1186/s12866-026-05390-0

**Published:** 2026-07-13

**Authors:** Wen Cui, Chuanmin Ma, Xichen Wang, Jishan Zhang, Lin Zhou, Jian Li, Xia Luo, Hui Sun, Xuefang Xu, Hui Liu

**Affiliations:** 1 Jinan Center for Disease Control and Prevention Affiliated to Shandong University, Jinan, Shandong 250021 China; 2 College of Traditional Chinese Medicine, Jinggangshan University, Ji’an, Jiangxi 343009 China; 3 National Key Laboratory of Intelligent Tracking and Forecasting for Infectious Diseases, National Institute for Communicable Diseases Control and Prevention, Chinese Center for Disease Control and Prevention, Changping, Beijing, China; 4 Shanghe County Center for Disease Control and Prevention, shanghe, Jinan, Shandong 250000 China

**Keywords:** *Clostridium botulinum*, Foodborne botulism, BoNT, Homemade pickled eggs, Subtype B2

## Abstract

Foodborne botulism (FB) results from the ingestion of food contaminated with botulinum neurotoxin. Here, we report a family outbreak of foodborne botulism caused by *Clostridium botulinum* subtype B2 linked to homemade pickled eggs. On August 13, 2024, the Jinan Center for Disease Control and Prevention and the Shanghe County Center for Disease Control and Prevention collaborated to investigate an outbreak of botulism poisoning related to a family gathering in Shanghe County, Jinan City. A total of 4 people attended the family gathering, three of whom developed clinical symptoms of botulism after consuming pickled eggs. The diagnosis of botulism was suspected through collaborative efforts by the Qilu Medical Prevention and Control Innovation Integration Mechanism and multidisciplinary consultations across multiple hospitals. We detected the *bont* gene using real-time quantitative PCR (qPCR) in Jinan CDC, determined the toxin serotype by mouse bioassay (MBA) in China CDC and performed whole-genome sequencing of bacterial strains isolated from patient feces, the homemade pickled eggs and soil in both laboratories. It was shown that the toxin type and *bont* gene were subtype B2, belonging to the *ha* gene cluster. The *Clostridium botulinum* strains isolated from two patients were closely related to the strains from the homemade pickled eggs through whole-genome single nucleotide polymorphism analysis. We also found that *Clostridium botulinum* strains from soil clustered into one branch with those from patients and food, suggesting that the source of *Clostridium botulinum* contamination may be from soil, although the contamination pathway was not clear. Importantly, our findings provided a basis for clinical antitoxin treatment, in which one patient successfully gave birth after recovery. These results underscore the importance of strengthening public education about the health risks associated with consuming homemade fermented or preserved foods and enhancing the laboratory detection capabilities.

**Supplementary Information:**

The online version contains supplementary material available at 10.1186/s12866-026-05390-0.

## Introduction

*Clostridium botulinum (C. botulinum)* is extensively spread in the natural environment, being commonly found in soil, sludge and animal feces [1, 2]. Generally, spores remain in a dormant state in the environment, but they can germinate and produce botulinum neurotoxins (BoNTs) under specific conditions such as anaerobiosis, certain pH value and low salt content and non-freezing temperatures. BoNTs can be produced not only by proteolytic and non-proteolytic *Clostridium botulinum*, but also by other Clostridia, such as *Clostridium butyricum* and *Clostridium baratii*. Normally, there are three common forms of botulism: foodborne botulism, infant botulism, and wound botulism [3–5]. In China, foodborne botulism is the predominant form of botulism. Ingestion of food contaminated with botulinum neurotoxins can lead to foodborne botulism [6]. Previous studies have demonstrated that even foods containing trace amounts of botulinum spores can multiply extensively in an appropriate environment and produce botulinum neurotoxin, leading to severe form of botulinum food poisoning [7].

BoNTs are one of the most toxic substances known to cause rare but serious neurological disorders [8, 9]. Clinical symptoms include nausea, vomiting, dizziness and generalized weakness, as well as more severe botulinum toxin-specific symptoms such as double vision, dysarthria, dysphonia, and dysphagia [10]. Botulinum neurotoxins are classically grouped into seven immunological serotypes, BoNT/A to BoNT/G. Each serotype can be further divided into sequence-defined subtypes based on genetic and amino-acid variation, such as BoNT/B1 and BoNT/B2. These groups differ in their toxin serotypes as well as in their growth requirements. Considerable research have demonstrated that all types of botulinum neurotoxin produce similar pharmacological effects, causing symptoms such as flaccid paralysis, but different types of toxins vary in terms of the duration and intensity of the symptoms [11, 12]. Normally, type A botulism has a shorter incubation period, more severe clinical symptoms and a faster progression rate, compared to other types [13]. Homemade pickled foods are the main cause of food-borne botulism. Nonetheless, it is worth noting that the majority of cases in China were caused by consuming raw pickled meat contaminated with botulinum neurotoxin, as well as homemade soy products and other foods [14]. Some studies have reported the distribution of main botulinum toxin subtypes varies significantly in different areas of China. This is mainly related to the regional distribution of *C. botulinum* in nature [14–17]. A previous study investigated *C. botulinum* contamination in the environmental surroundings of five botulism outbreaks in Heze, Shandong Province. The findings revealed that all *C. botulinum* strains detected in the environment were type B, consistent with the botulinum neurotoxin type responsible for the food poisoning incidents proving the local pollution [18]. This suggests that it is important to monitor the types of *C. botulinum* in the environment (soil) when tracing the source of botulism food poisoning events.

Here, we report a family outbreak of foodborne botulism. On August 13, 2024, the Jinan Municipal Center for Disease Control and Prevention received a report from the hospital that three patients had experienced blurred vision and difficulty in swallowing after consuming homemade pickled eggs. The clinical diagnosis was suspected to be botulism. To clarify the cause of poisoning and provide relevant support for clinical treatment, we conducted epidemiological investigation and collected food samples and patient fecal samples for subsequent laboratory testing in accordance with the GB/T 4789.12–2016 standard. Additionally, to further trace the potential source of contamination, soil samples were collected from the surrounding environment and subjected to laboratory analysis for the presence of *Clostridium botulinum* in China CDC. Meanwhile, the isolated bacterial strains underwent whole genome sequencing. Based on clinical symptoms, epidemiological investigation and laboratory test results and bioinformatics analysis, this incident was definitively diagnosed as foodborne botulism attributable to the consumption of homemade pickled eggs contaminated with the type B2 *C. botulinum* which was also detected in nearby soil. 

### Epidemiological investigation

The interviews with the patients revealed that three of them attended the family gatherings on August 3. A total of 4 people attended the family gathering, three of whom had clinical signs of botulism. The shared food included millet porridge, steamed buns, egg soup, cold noodles and homemade pickled eggs. After an epidemiological investigation, it was found that one person who had not consumed pickled eggs did not develop any symptoms. Therefore, homemade pickled eggs may be a risk food. Patient 1 (a pregnant woman) developed symptoms including fatigue and drooping eyelids on August 4. In the afternoon, she went to a county hospital and received consultations from the obstetrics department and the neurology department. She was given symptomatic treatment (without being hospitalized), but the symptoms did not improve. On August 7, patient 1 visited a provincial-level tertiary hospital but she was not diagnosed. Then, she returned to a county hospital for symptomatic treatment. On August 13, patient 1 was transferred to Shandong Provincial Third Hospital. Furthermore, on August 4, patients 2 and 3 experienced symptoms such as abdominal distension, blurred vision (double vision), drooping eyelids and difficulty in swallowing. They then received intravenous fluid replacement and supportive symptomatic care at the clinic to alleviate these symptoms. On August 12, botulism was suspected by the treating physician. The feces of patient 1 and patient 2 and homemade pickled eggs were collected and sent to the Microbiological Laboratory of the Jinan Center for Disease Control and Prevention on August 13. We did not obtain feces from patient 3 for unclear reasons. On August 15, hospital received the test results. Patient 1 was treated with heptavalent botulinum antitoxin (serotypes A-G) and was discharged on August 31 in stable condition. After follow-up, the baby has been born successfully and is in good health. The conditions of patients 2 and 3 improved after the antitoxin treatment. Informed consent was obtained from all subjects involved in the study. This study involving humans was reviewed and approved by the Public Health Ethics Committee of Shandong University, approval number: LL20220905.

### Traceback and investigation of the homemade pickled eggs

Patient 3 described the process of preparing homemade pickled eggs. Eggs were purchased from small vendors in the market. The process of making pickled eggs involves boiling the eggs, cracking the eggshells, adding brine (ratio 1:5), pickling them at room temperature in a sealed container for one week, changing the salt water and then refrigerating them for 10 days. The process of heating pickled eggs is missing before consumption. During the preparation process, the outdoor temperature exceeded 38℃. She described that this method was learned from friends and relatives. She also mentioned that she had not previously been aware of the risk of foodborne botulism associated with this approach.

## Materials and methods

### Sample enrichment and isolation of *C. botulinum*

The sample preparation and *Clostridium botulinum* isolation and identification were carried out in accordance with the method specified in GB 4789.12—2016 “National Food Safety Standard - Microbiological Examination of Food - *Clostridium botulinum* and botulinum neurotoxin Examination”. A total of 25 g of the homemade pickled eggs were added to 50 milliliters (ml) of gelatin phosphate buffer (GPB, pH 6.2, 2 g/L gelatin, and 4 g/L Na2HPO4) and then left to soak for 30 min. A volume of 2 ml of the sample homogenate or feces was inoculated separately into the cooked meat medium and TPGYT (China, Huankai) and anaerobically incubated at 36℃and 28℃, respectively, for 5 days to enrich for *C. botulinum*. The enriched culture medium was inoculated onto egg yolk agar (China, Huankai) and cultured anaerobically at 35℃ for 24 h. Suspicious colonies were selected and inoculated onto egg yolk agar again and then cultured anaerobically at 35℃ for 24 h to obtain pure colonies. The purified suspicious colonies were detected by Gram staining, fully automatic microbial mass spectrometry analyzer, real-time fluorescence PCR, mouse bioassay (MBA) and whole genome sequencing (WGS).

### Collection of soil samples from the vicinity of the patient’s residence and isolation of *C. botulinum*

We collected soil samples from the entrance of the residential unit where the poisoning cases resided, the four surrounding sides (east, south, west and north) of the building, the four corners of the community (southeast, northeast, southwest and northwest), and in four cardinal directions within a 5–10 km radius. A total of 26 soil samples were collected. The sampling method involved collecting composite samples from both the upper and lower soil layers at each site. The lower soil layer is collected at a depth of 10–20 cm, with a sample mass ranging from 100 to 200 g. All samples were placed in sterile sealed bags and labeled with the sampling location and time.

The collected soil samples were mixed with sterile water in a defined ratio to prepare soil suspensions at concentrations of 10^− 2^ to 10^− 6^. Soil dilutions of different concentrations were inoculated into cooked meat medium and TPGYT medium, respectively, and incubated at 36℃ for 5 to 10 days. Subsequently, the culture was streaked onto egg yolk agar and incubated at 35℃ for 48 h. Pure culture colonies were isolated and collected for relevant pathogen detection.

### DNA extraction and real-time PCR for BoNT gene detection

Homemade pickled eggs, two samples of feces and purified cultures were subjected to *bont* gene nucleic acid detection using real-time fluorescence quantitative PCR for rapid diagnosis. The total nucleic acid extraction was carried out using the QIAamp DNA Mini Kit (Germany, Qiagen) according to the manufacturer’s instructions. A 5 μL aliquot of nucleic acid was used as the template, and the C. botulinum toxin-producing gene detection kit (Shengkeyuan,China) was used to detect botulinum neurotoxin genes of types A, B, E, and F. Thermal cycling conditions consisted of a heat treatment at 50℃ for 2 min and a pre-denaturation step at 95℃ for 3 min, followed by 40 cycles at 95℃ for 3s and 55℃ for 30s. The measurements were completed with Thermofisher QuantStudio5 instrument (USA, Thermo Fisher Scientific).

### Mouse bioassay experiments

The 6–8-week-old female KM mice were raised for one week after purchase to adapt to the animal center environment with normal feeding and drinking at room temperature. The animals used in this study were obtained from Beijing Vital River Laboratory Animal Technology Co., Ltd, located in Beijing, China. The sample culture medium was centrifuged at 14,000×g for 5 min to remove the solid precipitation. The supernatant was divided into two equal portions, namely the supernatant group and the trypsin treatment group. All the mice were randomly divided into two groups: the supernatant group and the trypsin treatment group, with three mice in each. Each mouse was intraperitoneally injected with 0.5 ml of the corresponding preparation. The poisoning manifestations of the mice within 48 h were observed and recorded. Symptoms such as raised hair, dyspnea, weakness in the limbs and bellows-like breathing were observed. Based on these signs, it was preliminarily determined that it was caused by botulinum neurotoxin. Toxin-positive culture supernatants were subjected to a mouse neutralization assay. Equal volumes of culture supernatant and serotype-specific botulinum antitoxin were mixed and incubated at room temperature for 30 min before intraperitoneal inoculation into mice. Serotype-specific antitoxins against BoNT/A, BoNT/B, BoNT/E, and BoNT/F were obtained from China CDC (Beijing, China). The animals were euthanized by carbon dioxide (CO_2_) inhalation in a CO_2_ dedicated euthanasia chamber (Model: SMQ-II; Shanghai MinlyLab Hi-Tech Development Co.,Ltd). CO_2_ was introduced gradually at a displacement rate of 30–70% of the chamber volume per minute according to the approved animal ethics protocol until the animals became unconscious, as indicated by loss of the righting reflex and lack of response to external stimuli. CO_2_ exposure was maintained until respiration ceased. Death was confirmed by the absence of respiration, heartbeat, and reflexes. All animal experiments were performed in accordance with the Chinese Regulations on the Administration of Laboratory Animals and were approved by the Laboratory Animal Ethics Committee of the Chinese Center for Disease Control and Prevention (Approval Code: 2023-041; Approval Date: 18-10-2023).

### Whole genome sequencing of *C. botulinum* strains

Whole-genome sequencing (WGS) and genome characterization were conducted for thirteen putative *Clostridium botulinum* strains obtained from two clinical specimens (JNP1, JNP2), the homemade pickled egg sample (JNHPE) and ten soil samples (JNS1-JNS10). Sequencing was performed on the Illumina HiSeq 2500 platform (Illumina, San Diego, CA, USA) using a 150 bp paired-end (PE150) strategy. The raw reads were first processed with fastp (v0.23.4) to remove low-quality reads, reads containing excessive ambiguous bases, and adapter-contaminated reads. The resulting clean reads were de novo assembled using SOAPdenovo (v2.04), SPAdes (v3.10.0), and ABySS (v1.5.2). Assemblies generated by the three assemblers were integrated using CISA (v1.3), and the assembly with the fewest scaffolds was selected. GapCloser (v1.12) was subsequently applied to fill assembly gaps and improve assembly continuity. Contigs shorter than 500 bp were removed from the final assemblies prior to downstream analyses. The assembled genomes had average sequencing depths ranging from 288.16× to 367.06× and N50 values ranging from 29,597 to 2,087,955 bp, indicating sufficient sequencing coverage and variable assembly contiguity among the genome assemblies. Representative *Clostridium botulinum* genomes were selected from NCBI based on phylogenetic cluster, toxin serotype, geographical origin, botulism form, genome quality, and metadata availability. For phylogenetic inference based on core single-nucleotide polymorphisms (SNPs), contigs from these thirteen isolates together with 20 publicly available genomes retrieved from NCBI were aligned against the reference genome of *C. botulinum* strain B2 128 (GCF_000711065.1) [19] using Snippy (v4.6.0). *C. botulinum* strain B2 128 is a BoNT/B2-producing strain. A core genome alignment was generated using snippy-core, excluding non-core genomic regions, insertions/deletions, and low-quality sites. Finally, a maximum-likelihood (ML) phylogenetic tree was inferred in FastTree (v2.1) under the generalized time-reversible (GTR) model using the *coresnps.aln* alignment generated by snippy-core as input.

### Phylogenetic studies based on the bont gene sequence of botulinum neurotoxin

We accurately extracted the sequence of the *bont* gene from the whole genome sequencing results of the isolated *Clostridium botulinum* strains to clarify the evolutionary kinship and sequence characteristics of the *bont* gene among various subtypes of the isolated strains. The obtained *bont* sequences were subjected to multiple sequence alignment (MUSCLE) and homology comparison with the reference toxin sequences of the known B1-B8 subtypes. A high-resolution phylogenetic tree was constructed using the MEGA software (version 11.0.13) with the neighbor-joining method.

### Collinearity analysis reveals the structural characteristics of the toxin gene cluster

Furthermore, we analyzed the *bont* gene cluster encoding botulinum neurotoxin in the JNP1 isolate to further dissect the structural characteristics of the botulinum neurotoxin gene cluster. In addition, three representative sequences of botulinum neurotoxin gene clusters were obtained from the GenBank database as references. Subsequently, the collinearity analysis of the above-mentioned gene clusters was conducted using the Easyfig software (version 2.2.5, Brisbane, Australia), and the similarities and differences in gene arrangement, orientation and composition among the gene clusters were systematically compared.

### Antibiotic resistance genes and virulence factors of *C. botulinum*

The Prokka software was used to perform gene annotation on it to obtain the gene prediction results, and then the results were compared with the virulence factor database (VFDB) and the antibiotic resistance databases (CARD, ResFinder, ARGANNOT) to analyze whether there were virulence genes and antibiotic resistance genes in the strain.

## Results

### Isolation of *C. botulinum*

**Fig. 1:**
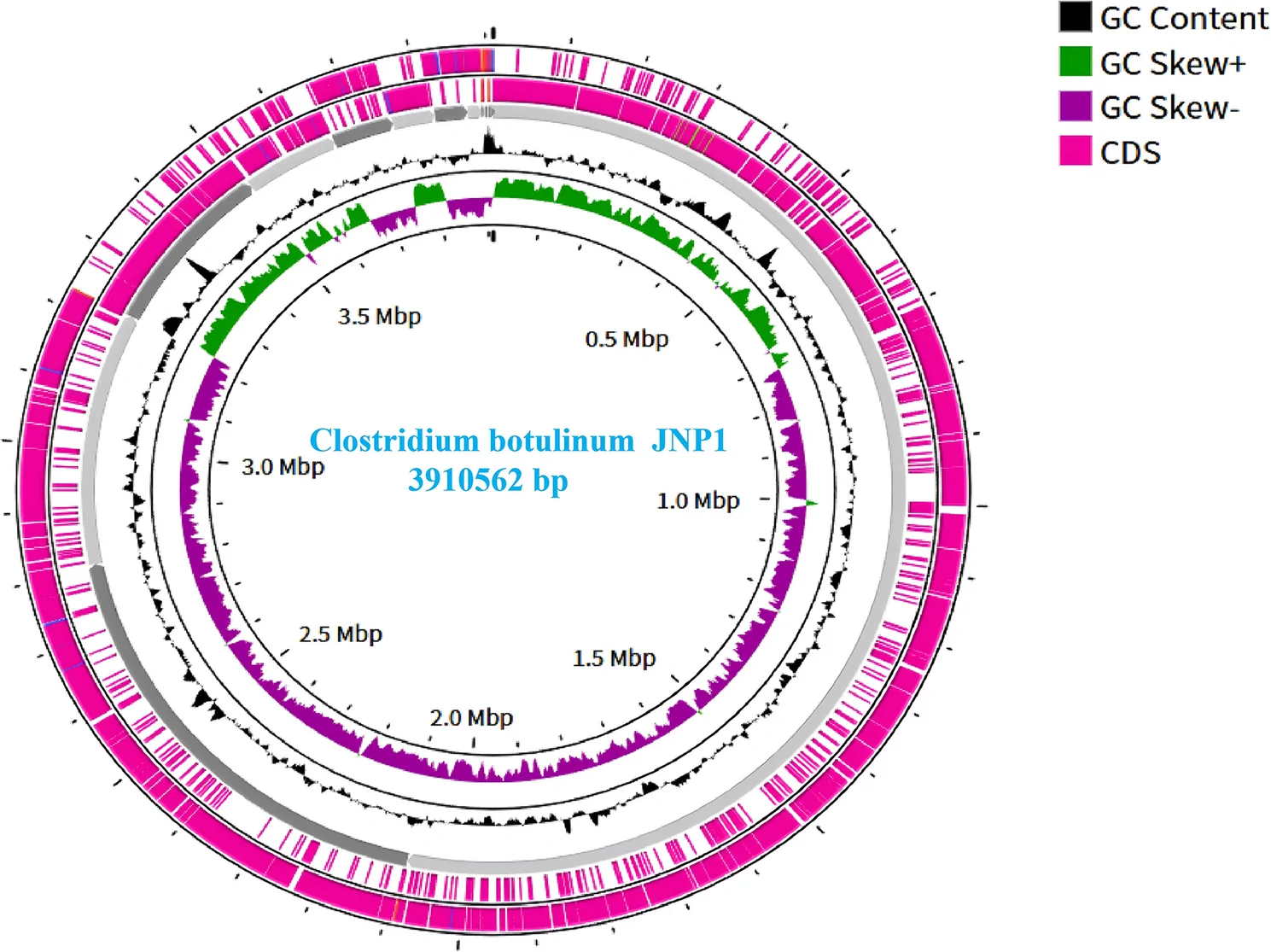
Circular map of the genome of *Clostridium botulinum* isolated JNP1 strain

Six *Clostridium botulinum* strains were isolated and confirmed by Gram staining and fully automatic microbial mass spectrometry analysis. Among them, one strain was from homemade pickled eggs (JNHPE), five strains were from the feces of patient 1 (JNP1) and patient 2 (JNP2) and soil (JNS1, JNS6, JNS8). Other strains (JNS2-5, JNS7, JNS9, JNS10) isolated from the soil were identified as *C. sporogenes* which is similar to *C. botulinum* in 16S rRNA and other phenotypic traits such as colony form and shape. The JNP1 sequencing results are shown in Fig. 1, with GC content in black and GC bias in green and purple. The genome sizes of JNHPE, JNP1, JNP2, JNS1, JNS6 and JNS8 were 3,864,574 bp, 3,910,562 bp, 3,795,124 bp, 3,935,412 bp, 3,914,578 bp and 3,836,476 bp, respectively. The GC contents were 28.15%, 28.07%, 27.63%, 28.58%, 27.32% and 27.87%, respectively.

### Detection of toxin genes and botulinum neurotoxin in samples

The homemade pickled eggs, two patient fecal samples and six isolated strains diluted with gelatin phosphate buffer solution were used for detection of four types of botulinum neurotoxin genes (A, B, E and F) by QIAamp DNA Mini Kit (Germany) nucleic acid extraction and fluorescence PCR analysis system. The results showed that all samples were bont B gene positive, since a typical S-shaped amplification curve was obtained for each one of them for type B **(**Table 1**)**. Supernatants were collected from the six samples after enrichment in cooked meat broth and TPGYT enrichment broth. The supernatant of the enrichment solution was directly injected into the abdominal cavity of mice. One hour after the injection, the miceed Meat Culture broth and TPGYT enrichment bro exhibited symptoms of botulism such as piloerection, weak limbs, and breathing difficulties. All of them died within 4 h. The supernatants of the above samples was treated with trypsin and then injected intraperitoneally into mice. The mice also exhibited symptoms of botulism and all died within 24 h. After further confirmation and typing tests for the toxin, it was confirmed that botulinum neurotoxin was detected in these samples and the toxin type was confirmed as type B (Table 1).

**Table 1:** Samples and test results

Source	qPCR	qPCR after enrichment	MALDI-TOF	Isolated strain	WGS	MBA
Patient 1 feces	*bont/B*	*bont/B*	*C. botulinum*	JNP1	BoNT/B2	BoNT/B
Patient 2 feces	*bont/B*	*bont/B*	*C. botulinum*	JNP2	BoNT/B2	BoNT/B
Homemade picked egg	*bont/B*	*bont/B*	*C. botulinum*	JNHPE	BoNT/B2	BoNT/B
Soil 1	ND	*bont/B*	*C. botulinum*	JNS1	BoNT/B2	BoNT/B
Soil 6	ND	*bont/B*	*C. botulinum*	JNS6	BoNT/B2	BoNT/B
Soil 8	ND	*bont/B*	*C. botulinum*	JNS8	BoNT/B2	BoNT/B

*Abbreviation: qPCR* Quantitative polymerase chain reaction, *bont/B* Botulinum neurotoxin/B, *ND* Not detected, *WGS* Whole genome sequencing, *MBA* Mouse Bioassay

### Phylogenetic analysis of *C. botulinum*

**Fig. 2:**
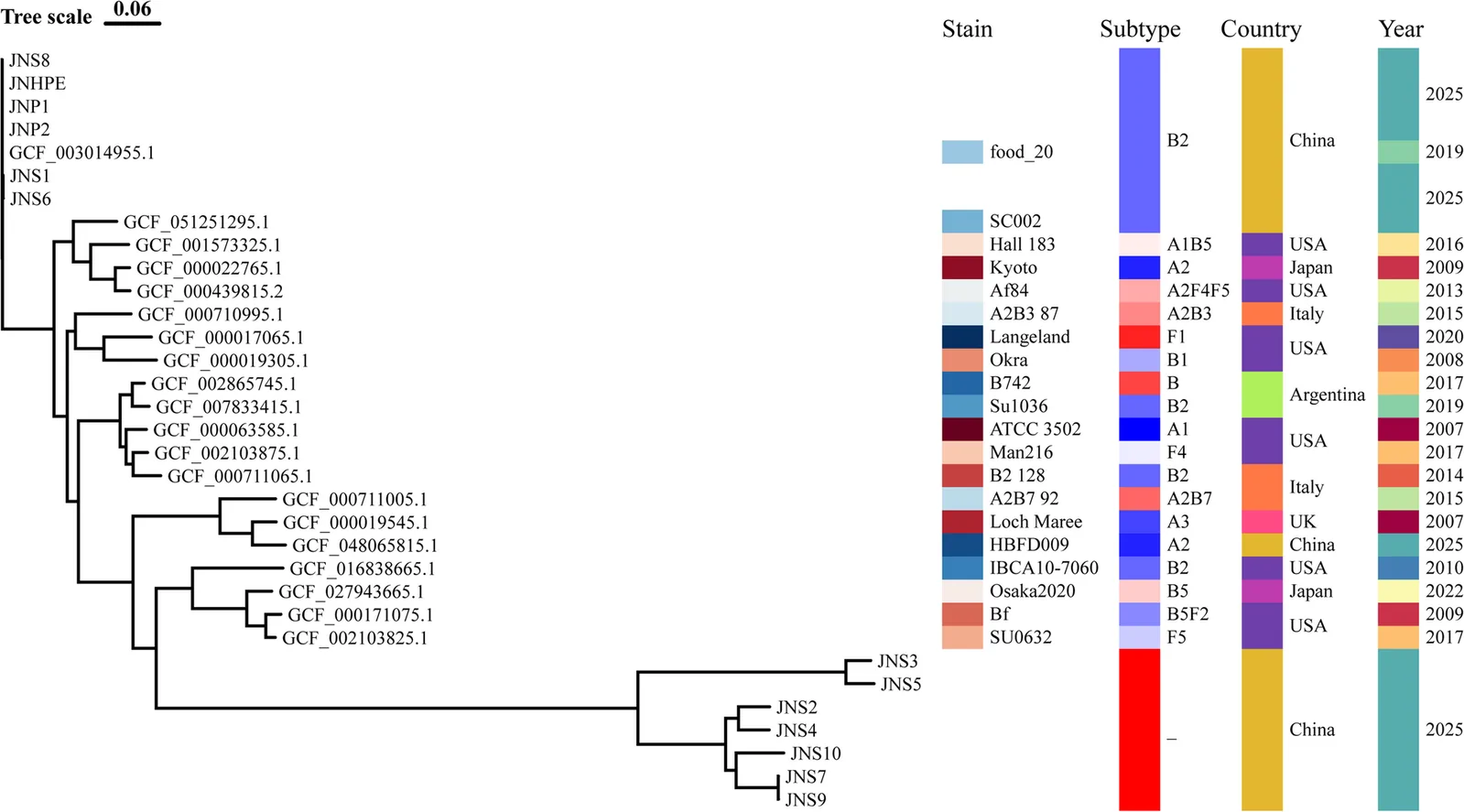
Construction of a core SNP-based phylogenetic tree of isolated *Clostridium botulinum*

Average nucleotide identity (ANI) analysis showed that the identity of chromosomal genome of JNHPE, JNP1, JNP2, JNS1, JNS6 and JNS8 were 97.69%, 97.35%, 97.84%, 97.16%, 98.12%, 97.89% with the reference sequence ATCC3502 of *Clostridium botulinum*, confirming that JNHPE, JNP1, JNP2, JNS1, JNS6 and JNS8 were *Clostridium botulinum*. Whole genome sequencing results were uploaded to the genome server (TYGS server), and the results showed that the isolated strains were identified as *C. botulinum*. We uploaded the complete genome sequence of *C. botulinum* isolates to the NCBI database (BioProject: PRJNA1450826). The phylogenetic tree based on 228,677 core SNPs showed that the six strains isolated from feces, soil and food belonged to Group I and formed a distinct cluster (Fig. 2). This indicated that these six strains were most likely from the same source.

### Botulinum neurotoxin subtypes

**Fig. 3:**
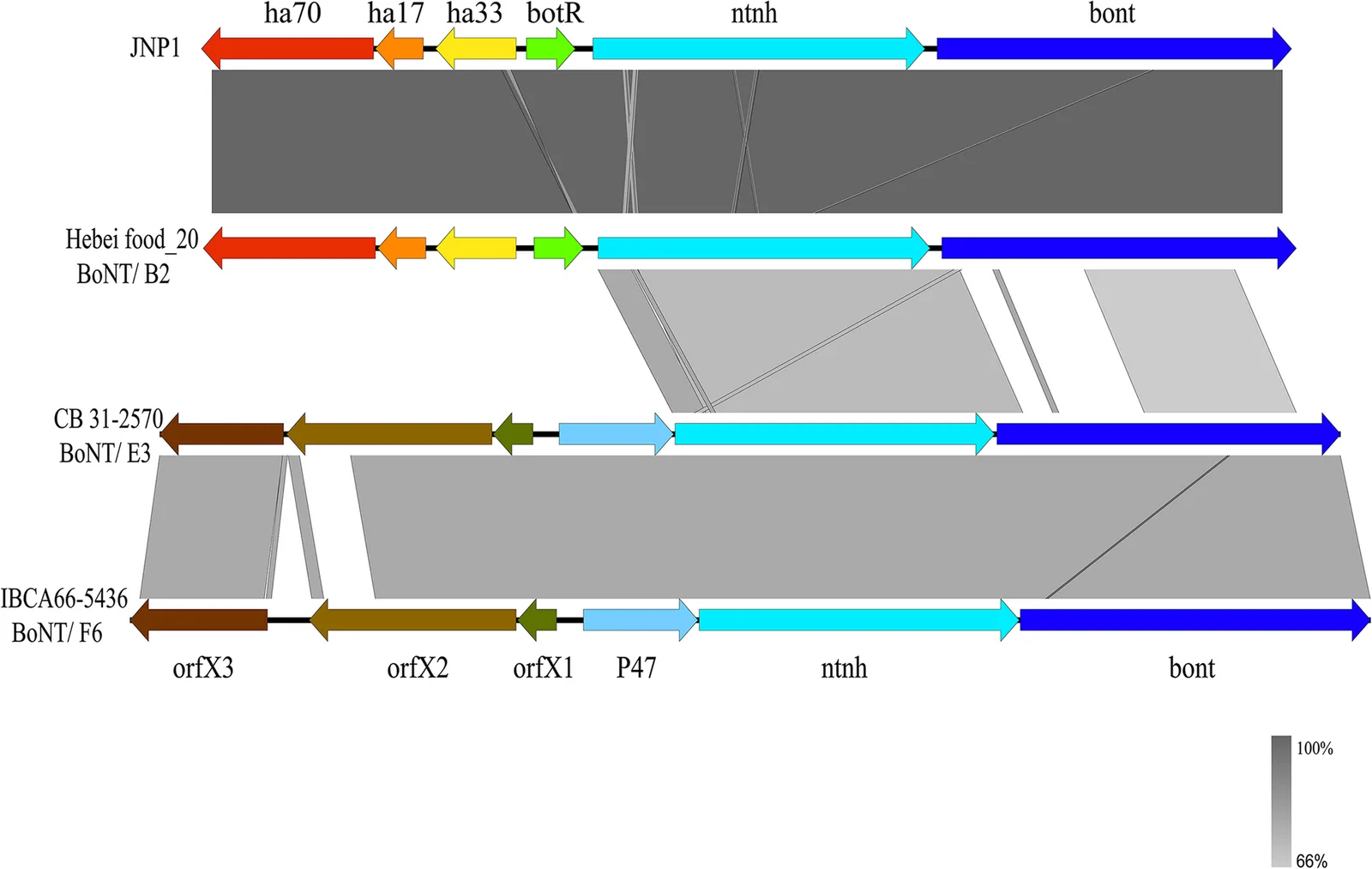
Comparison of collinearity analysis of toxin gene clusters in JNP1 and three subtypes of *Clostridium botulinum* (The gradient of gray levels represents different BLAST identity values)

**Fig. 4:**
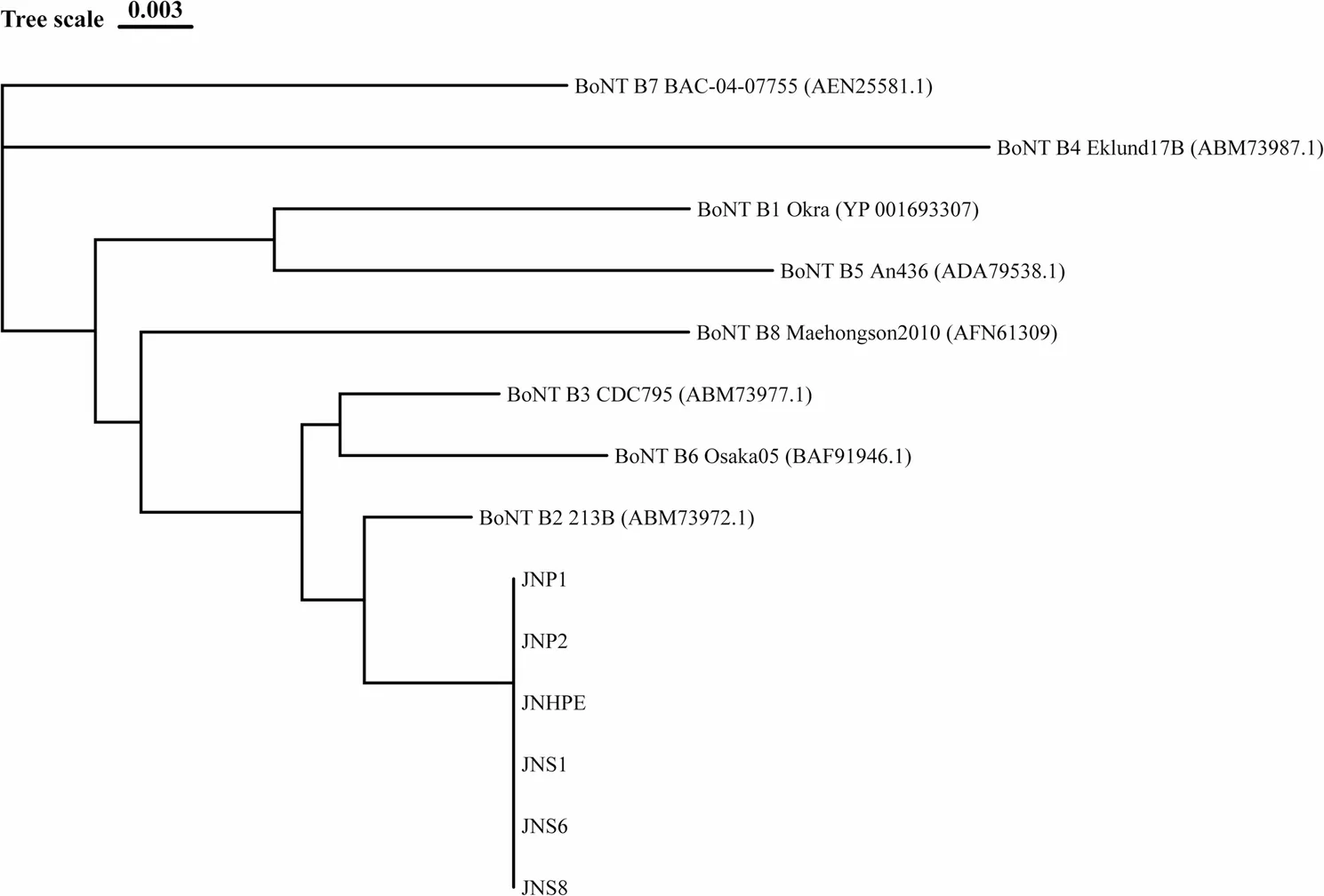
Phylogenetic tree constructed based on the nucleic acid sequence of the *bont* gene. (The accession number of each BoNT B reference sequence is indicated in parentheses after the subtype and strain name to specify the exact sequence used for comparison.)

Collinearity analysis with Easyfig (version 2.2.5) software showed that the toxin gene cluster of JNP1 had the same arrangement as BoNT/B2 of Hebei food_20 strain (Fig. 3). Blast results showed that the nucleotide sequence of *bont* had the highest similarity with BoNT/B2 subtype. The amino acid dissimilarity was below 2.6%, meeting the subtype B2 classification criterion [13]. Taken together, the results confirmed that the JNP1 strain belonged to the typical BoNT/B2 subtype. In addition, the results of the phylogenetic tree of *bont* gene showed that the *bont* gene of the isolated strains formed a branch with the B2 subtype, and the evolutionary relationship with other B subtypes was shown in the Fig. 4 .

### Neurotoxin gene clusters and MLST typing

The *botR*, *ha17*, *ha34* and *ha70* genes were found to be present in the JNP1 toxin gene cluster by Bakta software, indicating that the toxin gene was located within an hemagglutinin (HA) complex (Fig. 3). The ha cluster contains two transcription units. Genes encoding neurotoxin (*bont)* and nontoxic nonhemagglutinin (NTNH) are located in one transcription unit. Genes encoding hemagglutinins (*ha34*,* ha17* and *ha70*) are located in the other transcription unit, which transcribe in opposite directions. Multilocus sequence typing (MLST) analysis showed that all the isolated strains had the same alleles of the seven housekeeping genes: aceK18, aroE13, hsp11, mdh8, oppB9, recA10 and rpoB11, which were confirmed to be ST32.

### Virulence factors and antibiotic resistance genes

VFDB and CARD database were used to analyze the virulence genes and antibiotic resistance genes of the sequencing results, respectively. Eight virulence genes were identified, including *bont/b*, *htpB*, *clpP*, *glf*, *fliP*, *cloSI*, *pseB* and *clpC*. Among them, *bont/b* showed the highest sequence identity to known toxin gene references in VFDB. Six antibiotic resistance genes were identified, including *catB*, *vat(D)*, *ant(9)-Ia*, *cfr*, *lsa(E)* and *nimJ*. The sequence-identity analysis showed that *vat(D)* had the highest similarity to the reference resistance-gene sequences in the database.

## Discussion

Botulism is an acute paralytic disease caused by neurotoxins produced by *C. botulinum* and other BoNT-producing clostridia [5, 20]. Botulinum neurotoxin paralyses cranial nerves first and skeletal muscles later, and untreated poisoning can lead to severe flaccid paralysis, respiratory failure and death. Recognizing the early signs and symptoms of botulism can be challenging [21]. *C. botulinum* spores are ubiquitous, and improper preservation methods can promote spore germination and growth and BoNT production [22, 23]. Foodborne botulism occurs after the consuming food containing botulinum neurotoxin. In China, foodborne botulism mainly occurs in the northern provinces, especially in Qinghai, Xinjiang, Xizang and Hebei Provinces. It has been reported that homemade foods are the primary source of foodborne botulism in China [14, 17, 24]. This study investigates a household cluster of foodborne botulism poisoning incident in Jinan City, Shandong Province, caused by homemade pickled eggs contaminated with *C. botulinum* type B2. A total of three patients were identified. Two patients had mild symptoms and recovered after treatment. The pregnant woman had severe respiratory paralysis, which improved significantly after symptomatic and supportive treatment. She gave birth naturally and healthily during follow-up. Moreover, *Saravani* reported a case of a pregnant woman at 32 weeks gestation who suffered from severe foodborne botulism after consuming homemade dairy products [25]. That patient was successfully delivered by natural birth. Notably, the study found no adverse effects of botulism on pregnancy, delivery or fetal development, which is consistent with our data. It has been reported that this is mainly related to the inability of botulinum neurotoxin to cross the placental barrier and enter the fetal circulation. The direct impact of maternal botulism on fetal development appears to be minimal, based on current clinical evidence [26, 27]. However, severe complications (such as autonomic nervous dysfunction, respiratory failure, etc.) after poisoning in pregnant women can cause indirect damage to the fetus, such as hypoxia. Therefore, patients with suspected botulism during pregnancy should receive botulinum antitoxin treatment as soon as possible [27, 28].

**Fig. 5:**
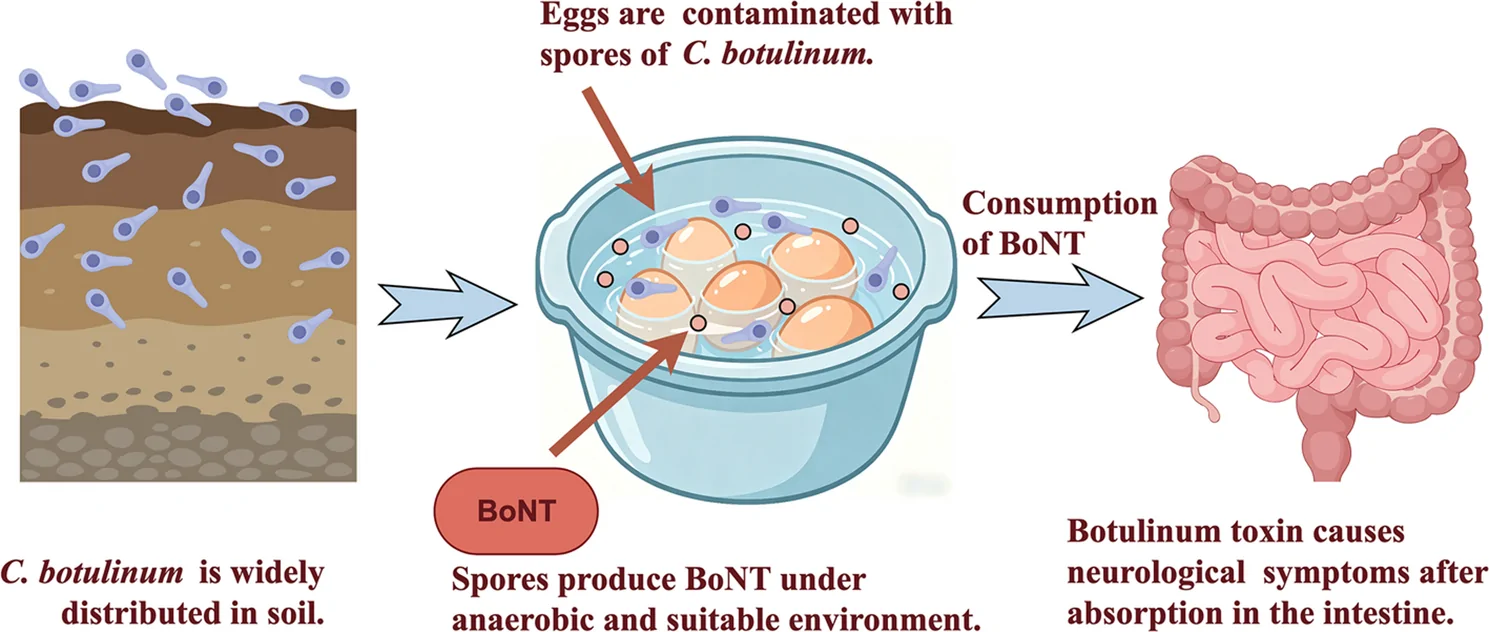
Eggs receiving *Clostridium botulinum* contamination from soil: implications for food poisoning (by figdraw.com)

In this study, home-made pickled eggs were identified as the most likely source of the outbreak. The epidemiological investigation showed that the homemade pickled eggs were prepared by boiling eggs, cracking the eggshells, pickling them in salted water at room temperature for two weeks, and then consuming them directly. During this process, the botulinum spores entered the egg through cracks and breaks in the shell, while the sealed pickling process formed an anaerobic environment that was conducive to spore recovery. In addition, the salt concentration of the eggs may not have reached a level that would inhibit *C. botulinum* spore germination, growth, and toxin production. As a high-protein food, eggs provide nutrition for the growth and reproduction of *C. botulinum*. The metabolism of *C. botulinum* and the production of toxin were accelerated by salting at room temperature. Collectively, these three factors led to the revival and massive proliferation of *C. botulinum*, resulting in toxin generation and subsequent foodborne botulism (Fig. 5). This suggests that we should strictly disinfect, control reasonable temperature and pH value, and boil before consumption when making homemade pickled high-protein food.

The primary homemade foods causing foodborne botulism in China include home-fermented products (stinky tofu, fermented bean paste), vacuum-packaged foods (spicy rabbit heads, salted fish), and air-dried meat products (beef jerky) [6, 14, 29, 30]. In Canada, food poisoning incidents are most commonly associated with traditionally processed fish products, whereas in Italy, they are most frequently linked to the consumption of canned vegetables (peppers, turnips) [31, 32]. In China, the most common toxin types causing foodborne botulism are type A (77.4%), type B (14.9%), and type E (5.8%) [33]. Type A is highly prevalent in Xinjiang [6]. In addition, it has been reported that the detection rate of *Clostridium botulinum* neurotoxin type A genes by whole genome sequencing and molecular identification was high in Sichuan Province, southwest China, from 1990 to 2024 [16]. Type B was highly prevalent in Hebei, Shandong and Henan provinces of the North China Plain [14]. Epidemiological data on botulism in Qinghai Province (1959–2022) indicated that type E botulism was the predominant form, primarily linked to the consumption of homemade air-dried meat and spoiled meat products [34]. Hence, differences in dietary habits and environmental factors across regions result in varying foods causing botulism poisoning. In 2000, the Centers for Disease Control and Prevention (CDC) in the United States reported the first case of botulism type B caused by consumption of homemade pickled eggs [35]. In 2024, a total of two cases of botulism caused by eating self-made pickled eggs were reported in China. One occurred in Weihai City, Shandong Province, involving botulinum neurotoxin type A3, while the second case was reported in Hebei Province, involving type A2 [36, 37]. Recent epidemiological studies also indicate that BoNT/B2 is an important subtype in both foodborne and infant botulism cases in Spain [38]. In this study, we isolated three strains of *Clostridium botulinum* from patient feces and eggs. Whole-genome sequencing and synteny analysis confirmed that all three strains were identified as BoNT/B2-producing *C. botulinum* strains. Phylogenetic tree analysis showed that the three *Clostridium botulinum* strains were closely related and clustered together, which, together with the epidemiological evidence, further supported homemade pickled eggs as the likely source of this outbreak.

*C. botulinum* is a spore-forming bacterium in soil and widely distributed. The detection rate of *C. botulinum* in soil was 22.2% in China, with the northwest region and the Qinghai-Tibet Plateau being the primary distribution areas. In 1922, *Shoenhol and Meyer* found the first strain of *C. botulinum* (type B) in soil in Hebei and Shanxi provinces in China [39]. Subsequently, *C. botulinum* was isolated from soil, marine sediment, and other samples in multiple regions across the country. Research reports indicate that investigations into *C. botulinum* contamination in soil, seafood, and grain products in Hebei Province revealed a detection rate of 2.8%, with type B being the predominant toxin type [40]. There have been few reports on *Clostridium botulinum* contamination in soils of Shandong Province. A survey conducted by Gao Qingyi et al. on *C. botulinum* contamination in coastal soils and various marine fish revealed that all serotypes (A, B, C, D, E, and F) were present in China’s natural environment, with type A predominating in the Weihai region of Shandong Province [41]. It was reported that *C. botulinum* type B predominates among strains isolated from soil in Heze City, Shandong Province [42]. In addition, all the strains isolated from the Weishan Lake area of Jining were BoNT/E-producing *Clostridium butyricum*, and the positive rate was as high as 36% [43]. In this study, we isolated *C. botulinum* from the soil of the patient’s residence and compared its genome sequences with those of strains from food and patients to facilitate further source tracing. A total of 3 strains of *C. botulinum* were isolated from soil, and the sequencing analysis identified all three isolates as BoNT/B2-producing *C. botulinum* strains. Seven *C. sporogenes* were also isolated from the soil. Previous studies have shown that some *C. sporogenes* strains can carry the *bont/B2* gene or produce BoNT/B2 [44]. Hence, genomes from three *C. botulinum* strains and seven *C. sporogenes* isolated from soil were used to construct a core SNP-based phylogenetic tree. *C. sporogenes* isolates recovered from soil were phylogenetically distant from the botulinum neurotoxin-producing strain associated with this event. However, the patient-derived isolate (JNP1) and the soil-derived isolates (JNS1, JNS6, JNS8) differed by only 24, 26, 22 core SNPs, respectively, indicating a very high level of genomic similarity. The *Clostridium botulinum* isolate JNS1 was obtained from both sides of the entrance to the residential building in which the patient lived, at the location closest to the patients residence. JNS6 and JNS8 were obtained from the northeast and southwest corners of the residential community, respectively. This result supports the inference that the soil surrounding the outbreak site could have been the source of contamination. The results indicate that the soil-derived *C. botulinum* strains share the same evolutionary branch with sequences from patient feces and homemade eggs, suggesting soil may be a potential source of contamination.

Interestingly, the *C. botulinum* strain we isolated from soil belongs to the same evolutionary branch as the Hebei strains isolated from infant botulism cases in 2019. In 2019, an infant botulism outbreak was reported. Tracing the source revealed that contaminated food came from an opened rice cereal container. *C. botulinum* strains food_20 and feces_22 were isolated from the food and stool samples, respectively. As environmental testing was not performed, it is not clear how the opened container became contaminated with *C. botulinum* [45]. Our results also provided evidence for the environmental traceability process of infant botulism in Hebei Province. The survival and distribution of *C. botulinum* spores in soil are influenced by environmental factors. Shandong Province and Hebei Province both located in North China Plain, with similar climate type conditions (temperate monsoon climate), soil texture (such as alluvial soil of the Yellow River), humidity, temperature and other environmental factors. Some studies have reported that the geographical distribution characteristics of botulism outbreaks are consistent with the distribution characteristics of *C. botulinum* in soil [46]. It is suggested that if a poisoning case is found, the possibility of cross-provincial contamination should be considered when tracing the source of the strain, and cross-regional cooperation is needed for prevention and control. The genes encoding botulinum neurotoxin proteins occur in two distinct toxin gene clusters: the *orfX* toxin gene cluster and the hemagglutinin (ha) toxin gene cluster [47]. The *orfX* toxin gene cluster contains one non-toxin non-hemagglutinin (*ntnh*) gene, one regulatory gene (*botR*), one gene of unknown function (*p47*), and three predicted open reading frame gene clusters orfX1-X3 [48]. The hemagglutinin (ha+) toxin gene cluster contains three hemagglutinin genes (*ha70*,* ha17*,* ha33*) in place of the *orfX1-X3* genes and lacks the *p47*gene [49]. Toxin gene clusters are associated with their specific toxin subtypes. In this study, a total of six *C. botulinum* isolates were identified as BoNT/B2 subtype. Collinearity analysis confirmed that the genes encoding B2 toxin were localized on the hemagglutinin complex (ha) containing *ha70*, *ha17*, *ha33*, *botR*, *ntnh* and *bont/B2* genes, which was consistent with previous studies. 

In conclusion, all three patients were recovered and improved markedly after antitoxin treatment including one pregnant woman. Comprehensive analysis of the patients’ clinical symptoms, epidemiological investigation results and laboratory test results showed that the outbreak was a foodborne botulism outbreak caused by homemade pickled eggs contaminated with BoNT/B2. This outbreak underscores the need to enhance public health education in rural areas regarding the risks of foodborne botulism associated with consuming homemade pickled eggs. Individuals experiencing similar symptoms should seek medical attention promptly. Additionally, diagnosing botulism is challenging and prone to misdiagnosis or delayed diagnosis, necessitating enhanced clinical diagnostic capabilities among physicians which can be improved by training and supported by laboratory detection.

## Supplementary Information

Supplementary Material 1.

Supplementary Material 2.

## Data Availability

Publicly available in a repository: The raw sequencing data generated in this study have been deposited in the NCBI Sequence Read Archive under BioProject accession number PRJNA1450826, with accession numbers SRR38017220, and are publicly available at https://www.ncbi.nlm.nih.gov/bioproject/PRJNA1450826.

## Abbreviations

FB: Foodborne botulism
MBA: Mouse bioassay
*C. botulinum*: Clostridium botulinum
BoNTs: Botulinum neurotoxins
ml: Milliliters
WGS: Whole genome sequencing
CO2: Carbon dioxide
SNPs: Single-nucleotide polymorphisms
ML: Maximum likelihood
GTR: Generalized time-reversible
qPCR: Quantitative polymerasechain reaction
BoNT/B: Botulinum neurotoxin/B
ND: Not detected
NTNH: Nontoxic nonhemagglutinin
ha: Hemagglutinin

## References

[1] Espelund M, Klaveness D. Botulism outbreaks in natural environments - an update. Front Microbiol. 2014;11:287. 10.3389/fmicb.2014.00287 PMC4052663 24966853

[2] Brunt J, van Vliet AHM, Stringer SC, Carter AT, Lindström M, Peck MW. Pan-Genomic analysis of *Clostridium botulinum* group II (Non-Proteolytic *C. botulinum*) associated with foodborne botulism and isolated from the environment. Toxins. 2020;12:306. 32397147 10.3390/toxins12050306 PMC7291236

[3] Raatjes GJ, Smelt JP. *Clostridium botulinum* can grow and form toxin at pH values lower than 4.6. Nature. 1979;281:398–9. 39257 10.1038/281398a0

[4] Sobel J, Botulism. Clin Infect Dis. 2005;41:1167–73. 16163636 10.1086/444507

[5] Rawson AM, Dempster AW, Humphreys CM, Minton NP. Pathogenicity and virulence of *Clostridium botulinum*. Virulence. 2023;14:2205251. 37157163 10.1080/21505594.2023.2205251 PMC10171130

[6] Ma X, Li KX, Li F, Su J, Meng WW, Sun YM, et al. Tracing foodborne botulism events caused by *Clostridium botulinum* in Xinjiang Province, China, using a Core genome sequence typing scheme. Microbiol Spectr. 2022;10:e0116422. 36377961 10.1128/spectrum.01164-22 PMC9769928

[7] Bowe BK, Wentz TG, Gregg BM, Tepp WH, Schill KM, Sharma SS, et al. Genomic diversity, competition, and toxin production by Group I and II *Clostridium botulinum* strains used in food challenge studies. Microorganisms. 2022;10:1895. 36296172 10.3390/microorganisms10101895 PMC9611418

[8] Pirazzini M, Rossetto O, Eleopra R, Montecucco C. Botulinum neurotoxins: biology, pharmacology, and toxicology. Pharmacol Rev. 2017;69:200–35. 28356439 10.1124/pr.116.012658 PMC5394922

[9] Monash A, Tam J, Rosen O, Soreq H. Botulinum neurotoxins: history, mechanism, and applications. a narrative review. J Neurochem. 2025;169:e70187. 40762356 10.1111/jnc.70187 PMC12323300

[10] Rao AK, Sobel J, Chatham-Stephens K, Luquez C. Clinical guidelines for diagnosis and treatment of botulism, 2021. MMWR Recomm Rep. 2021;70:1–30. 33956777 10.15585/mmwr.rr7002a1 PMC8112830

[11] Chatham-Stephens K, Fleck-Derderian S, Johnson SD, Sobel J, Rao AK, Meaney-Delman D. Clinical features of foodborne and wound botulism: a systematic review of the literature, 1932–2015. Clin Infect Dis. 2017;66:S11–6. 29293923 10.1093/cid/cix811

[12] Brunt J, van Vliet AHM, Carter AT, Stringer SC, Amar C, Grant KA, et al. Diversity of the genomes and neurotoxins of strains of *Clostridium botulinum* group I and *Clostridium sporogenes* associated with foodborne, infant and wound botulism. Toxins. 2020;12:586. 32932818 10.3390/toxins12090586 PMC7551954

[13] Carter AT, Peck MW. Genomes, neurotoxins and biology of *Clostridium botulinum* Group I and Group II. Res Microbiol. 2015;166:303–17. 25445012 10.1016/j.resmic.2014.10.010 PMC4430135

[14] Li HQ, Guo YC, Tian T, Guo WH, Liu CQ, Liang XC, et al. Epidemiological analysis of foodborne botulism outbreaks - China, 2004–2020. China CDC Wkly. 2022;4:788–92. 36284604 10.46234/ccdcw2022.114 PMC9547723

[15] Lin YJ, Li F, Su J, Meng WW, Tian T, Yuan YH, et al. Tracing investigation and analysis of a *Clostridium botulinum* food poisoning incident in Bayingolin Mongolian Autonomous Prefecture, Xinjiang. Zhonghua Yu Fang Yi Xue Za Zhi. 2022;56:541–4. 35488556 10.3760/cma.j.cn112150-20220118-00066

[16] Liu SY, Huang YL, Zhang L, Liu L, Zhao WX, Lei GP, et al. Molecular characterization of *Clostridium botulinum* isolates from sichuan province, China (1990–2024). Foodborne Pathog Dis. 2025;23:20–8. 40766982 10.1177/15353141251365831

[17] Fu SW, Wang CH. An overview of type E botulism in China. Biomed Environ Sci. 2008;21:353–6. 18837301 10.1016/S0895-3988(08)60054-9

[18] Li TB, Zhao XL, Zhang XS, Li QY, Chen SC, Zhang JJ. Diagnosis and treatment of botulism and investigation of contamination status. China public health. 2000;16:691–691.

[19] Peck MW. Biology and genomic analysis of *Clostridium botulinum* and related species. Genome Biol. 2009;10:R115.19835606

[20] Douillard FP, Derman Y, Woudstra C, Selby K, Mäklin T, Dorner MB, et al. Genomic and phenotypic characterization of *Clostridium botulinum* isolates from an infant botulism case suggests adaptation signatures to the gut. mBio. 2022;13:e0238421. 35499308 10.1128/mbio.02384-21 PMC9239077

[21] Cai S, Singh BR, Sharma S. Botulism diagnostics: from clinical symptoms to in vitro assays. Crit Rev Microbiol. 2007;33:109–25. 17558660 10.1080/10408410701364562

[22] Harris RA, Dabritz HA. Infant botulism: in search of *Clostridium botulinum* spores. Curr Microbiol. 2024;81:306. 39138824 10.1007/s00284-024-03828-0 PMC11322261

[23] Relun A, Dorso L, Douart A, Chartier C, Guatteo R, Mazuet C, et al. A large outbreak of bovine botulism possibly linked to a massive contamination of grass silage by type D/C *Clostridium botulinum* spores on a farm with dairy and poultry operations. Epidemiol Infect. 2017;145:3477–85. 29094676 10.1017/S0950268817002382 PMC9148736

[24] Gao QY, Huang YF, Wu JG, Liu HD, Xia HQ. A review of botulism in China. Biomed Environ Sci. 1990;3:326–36. 2252552

[25] Saravani K, Kamrava A, Poursamimi J. Familial cluster of foodborne botulism associated with homemade dairy products: a case series. Int Med Case Rep J. 2025;18:1295–301. 41089348 10.2147/IMCRJ.S553444 PMC12517297

[26] Malov VA, Tsvetkova NA, Baka KN, Volchkova EV, Konnova YA, Maleyev VV, et al. The problem of diagnosis and differential diagnosis of botulism in pregnant women. Case report. Terapevticheskii Arkh (Ter Arkh. 2021;93:1368–74. 10.26442/00403660.2021.11.201195 36286661

[27] Badell ML, Rimawi BH, Rao AK, Jamieson DJ, Rasmussen S, Meaney-Delman D. Botulism during pregnancy and the postpartum period: a systematic review. Clin Infect Dis. 2017;66:S30–7. 29293925 10.1093/cid/cix813

[28] Nair JJ, Balachandran DM. Botulism in pregnancy: a clinical review. Toxicon. 2025;267:108601. 41015266 10.1016/j.toxicon.2025.108601

[29] Min M, Bai LL, Peng XB, Guo L, Wan K, Qiu ZW. An outbreak of botulinum types A, B, and E associated with vacuum-packaged salted fish and ham. J Emerg Med. 2012;60:760–3. 10.1016/j.jemermed.2020.12.006 33518376

[30] Cui W, Ma CM, Liu M, Li Y, Zhou L, Shi YW, et al. Foodborne botulism caused by *Clostridium botulinum* subtype A5(b3) by self-packaged vacuum spicy rabbit heads. Microorganisms. 2025;13:1662. 40732171 10.3390/microorganisms13071662 PMC12298240

[31] Harris RA, Tchao C, Prystajecky N, Weedmark K, Tcholakov Y, Lefebvre M, et al. Foodborne botulism, Canada, 2006–2021. Emerg Infect Dis. 2023;29:1730–7. 37610295 10.3201/eid2909.230409 PMC10461667

[32] Giordani F, Fillo S, Anselmo A, Palozzi AM, Fortunato A, Gentile B, et al. Genomic characterization of Italian *Clostridium botulinum* group I strains. Infect Genet Evol. 2015;36:62–71. 26341861 10.1016/j.meegid.2015.08.042

[33] Zhan YS, Li WW, Yan SF, Ma X, Yang XR, Zhang M, et al. Contamination of *Clostridium botulinum* and epidemic characteristics of foodborne botulism in China. Chin J food hygiene. 2025;37:880–92.

[34] Wang YL, Guo XB. Epidemiological analysis of foodborne botulism outbreaks in Qinghai province from 1959 to 2022. Chin J Food Hygiene. 2024;36:207–11.

[35] Centers for Disease Control and Prevention. Foodborne botulism from eating home pickled eggs–Illinois, 1997. MMWR Morb Mortal Wkly Rep. 2000;49:778–80. 10987245

[36] Miao TT, Li X, Hu B, Zhang HJ, Xu L, Zhang DF, et al. An outbreak of foodborne botulism caused by *Clostridium botulinum* BoNT/A3 in pickled eggs - Weihai City, Shandong Province, China, July 2024. China CDC Wkly. 2024;6:1375–80. 39802083 10.46234/ccdcw2024.273 PMC11724129

[37] Lu ZW, Feng JZ, Luo X, Sun H, Huang Y, Lu SS, et al. Tracing and characterization of foodborne botulism caused by the new MLST type *Clostridium botulinum* A2 in Hebei province, China. Front Microbiol. 2025;16:1567360. 40207164 10.3389/fmicb.2025.1567360 PMC11979207

[38] Valdezate S, Carrasco G, Medina MJ, Garrido N, Del Pino S, Valiente M, et al. Exploring the genetic background of the botulism neurotoxin BoNT/B2 in Spain. Microbiol Spectr. 2023;11:e0238023. 37750689 10.1128/spectrum.02380-23 PMC10581064

[39] Wang ZY, Zheng ZR. Analysis of geographical distribution characteristics of *Clostridium botulinum* in China. Chin J Public Health. 1992;8:461–3.

[40] Hou ZZ. Preliminary report on the pollution of toxin-producing *Clostridium botulinum* in the natural environment of Hebei Province. Chin J Public Health. 1985;4:10–1.

[41] Gao QY, Liu HD, Yao JH, Wu JG, Chen XN, Dai CF, et al. Investigation on the pollution of toxin-producing Clostridium botulinum in soil and seafood in coastal areas of China. J Hygiene Res. 1983;12:28–32.

[42] Liu YG, Si PH, Zhang FC, Hou FY. Investigation and analysis of *Clostridium botulinum* pollution in the surrounding environment of botulism patients. Chin J Food Hygiene. 1994;6:43–4.

[43] Zou KY, Kuang HL, Meng XQ, Wang XM, Lu J, Wang CH. Investigation on the soil distribution of neurotoxinogenic *Clostridium tyrobutyricum* along Weishan Lake area. Progress Microbiol Immunol. 1997;25:6–10.

[44] Grenda T, Grenda A, Jakubczyk A, Rybczyńska-Tkaczyk K. Opportunistic features of non-*Clostridium botulinum* strains containing bont gene cluster. Pathogens. 2024;13:780. 39338971 10.3390/pathogens13090780 PMC11435427

[45] Xin WW, Huang Y, Ji B, Li P, Wu Y, Liu J, et al. Identification and characterization of *Clostridium botulinum* strains associated with an infant botulism case in China. Anaerobe. 2019;55:1–7. 30401636 10.1016/j.anaerobe.2018.06.015

[46] Hu YT, Tong XZ. Investigation on botulism and ecological environment in China. Chin J Epidemiol. 1988;9:171–6. 3064917

[47] Peck MW. Biology and genomic analysis of *Clostridium botulinum*. Advancesin Microb Physiol. 2009;55:183e265. 10.1016/S0065-2911(09)05503-9 19573697

[48] Raphael BH, Luquez C, McCroskey LM, Joseph LA, Jacobson MJ, Johnson EA, et al. Genetic homogeneity of *Clostridium botulinum* type A1 strains with unique toxin gene clusters. Appl Environ Microbiol. 2008;74:4390–7. 18502928 10.1128/AEM.00260-08 PMC2493146

[49] Amatsu S, Fujinaga Y. Botulinum hemagglutinin: critical protein for adhesion and absorption of neurotoxin complex in host intestine. Methods Mol Biol. 2020;2132:183–90. 32306327 10.1007/978-1-0716-0430-4_19

